# Are doctors using more preventive medication for cardiovascular disease? A Swedish cross-sectional study

**DOI:** 10.1080/02813432.2023.2234439

**Published:** 2023-07-19

**Authors:** Joel Lillqvist, Johan N. Sommar, Per E. Gustafsson, Eva-Lotta Glader, Katarina Hamberg, Olov Rolandsson

**Affiliations:** a Department of Public Health and Clinical Medicine, Umeå University, Umea, Sweden; b Department of Public Health and Clinical Medicine, Sustainable Health, Umeå University, Umea, Sweden; c Department of Epidemiology and Global Health, Umeå University, Umea, Sweden

**Keywords:** Healthcare inequities, pharmacoepidemiology, cardiovascular disease, prevention, epidemiology

## Abstract

**Background:**

Despite decreasing mortality from cardiovascular disease (CVD), there are persistent inequities in mortality between socioeconomic groups. Primary preventative medications reduce mortality in CVD; thus, inequitable treatments will contribute to unequal outcomes. Physicians might contribute to inequality by prescribing preventative medication for CVD to themselves in a biased manner.

**Aim:**

To determine whether primary medications for preventing CVD were prescribed inequitably between physicians and non-physicians.

**Design and setting:**

This retrospective study retrieved registry data on prescribed medications for all physicians in Sweden aged 45–74 years, during 2013, and for reference non-physician individuals, matched by sex, age, residence, and level of education. The outcome was any medication for preventing CVD, received at least once during 2013.

**Method:**

Age and the sex-specific prevalence of myocardial infarction (MI) among physicians and non-physicians were used as a proxy for the need for medication. Thereafter, to limit the analysis to preventative medication, we excluded individuals that were diagnosed with CVD or diabetes. To analyse differences in medication usage between physicians and matched non-physicians, we estimated odds ratios (ORs) with conditional logistic regression and adjusted for need and household income.

**Results:**

MI prevalences were 5.7% for men and 2.3% for women, among physicians, and 5.4% for men and 1.8% for women, among non-physicians. We included 25,105 physicians and 44,366 non-physicians. The OR for physicians receiving any CVD preventative medication, compared to non-physicians, was 1.65 (95% confidence interval 1.59–1.72).

**Conclusion:**

We found an inequity in prescribed preventative CVD medications, which favoured physicians over non-physicians.

## Summary

Groups with low socioeconomic status have lower rates of using medication that prevents cardiovascular disease, compared to groups with high socioeconomic status.

Physicians are responsible for prescribing all medicines to prevent cardiovascular disease; thus, biased prescriptions could have effects on the equality of care in the population.

Compared to individuals with equivalent education, physicians had higher rates of using medication that prevents cardiovascular disease.

This study highlights the need for systematic population-based evaluation of CVD risk in order to promote equitable CVD outcomes.

## 1. Introduction

Longevity has increased in Western society during the last 30 years. However, inequity in life expectancy persists among different socioeconomic positions (SEPs) in Europe, even in welfare states, like the Nordic countries [1,2]. For example, in 2018, life expectancy in Sweden at 30 years of age was 6 years longer for people with post-high school education compared to those with only pre-high school education [3]. A number of explanations for these inequities have been proposed: (1) there are substantial inequalities in the access to material and immaterial resources; (2) due to greater intergenerational mobility, the composition of lower socioeconomic groups has become more homogeneous, with regard to personal characteristics associated with ill-health; and (3) due to a change in epidemiology, where consumption behaviour has become the most important determinant of ill health, those in a higher SEP have increased access to the immaterial resources that provide support in making healthy life-style-choices [1]. Moreover, part of this inequity could be explained by findings that groups with lower SEP receive less healthcare than high-SEP groups, after adjusting for morbidity [4,5].

Horizontal inequity is when individuals with similar healthcare needs do not receive similar treatment [6]. When there is horizontal inequity in the prescription of preventative medications for cardiovascular disease (CVD), the inequity in CVD-related deaths is likely to increase [7]. Previous studies on the medical prevention of CVD have concluded that CVD risk is appropriately assessed and treated in a higher proportion of high-SEP individuals, compared to low-SEP individuals [8,9].

A reduction in CVD mortality contributes significantly to an increase in life expectancy [10]. In addition to improving acute medical treatment options, in the general population, CVD mortality can be reduced by improving risk factors, such as smoking, diet, physical activity, blood pressure, and cholesterol levels [11,12]. Cholesterol-lowering treatments and antihypertensive medications target the two greatest CVD risk factors that can be treated with medicines [11].

One example of horizontal inequity occurs when physicians evaluate their own or their colleagues CVD risk differently from how they evaluate CVD risk in patients. Physicians in Sweden, as in many of the Nordic countries, are able to prescribe medicines to themselves without consulting another physician, creating an opportunity for non-objective evaluations of the physician’s own CVD-risk. In a Norwegian study, it was reported that 73% of physicians using prescription medications were self-prescribing [13]. Horizontal inequity in prescribing primary preventative CVD medications, e.g. statins or antihypertensives, might contribute to an inequity in CVD-related deaths when physicians either overprescribe medicines for themselves or under-prescribe medicines for others. For example, it was shown in the UK that female general practitioners used hormone replacement therapy to a greater extent (∼40%) than non-physician women in the same age group (∼10%) [14]. However, physicians represent only a small fraction of the population. Nevertheless, a potential bias in prescriptions among physicians is an important example of horizontal inequity, because it could potentially translate into biased prescriptions for the general population because physicians are responsible for all prescriptions to the general public. In turn, that inequity could lead to inequitable health outcomes.

Previous studies on the use of medications among physicians were based on self-reported data or lacked appropriate controls [13–20]. To the best of our knowledge, no studies have investigated horizontal inequity in the use of statins and antihypertensives among physicians, compared to non-physicians. Thus, we aimed to investigate whether horizontal inequity in medical treatments for primary CVD prevention existed between physicians and non-physicians with equivalent education.

## 2. Methods

### 2.1. Population

This cross-sectional study included physicians and non-physician individuals, matched for sex, age, education, and area of residence. We extracted data from the Statistics Sweden registry on 28,524 physicians, aged 45–74 years, that were living in Sweden on 31 Dec 2013. Statistics Sweden tools allowed us to match each physician with one or two unique non-physicians of the same sex and with five or more years of university education. We first sought to identify reference individuals that lived in the same municipality as the physicians; when that was not possible, we sought individuals that lived in the same county as the physicians; and when that was not possible, reference individuals were drawn from anywhere in the country. The study was approved by the Regional Ethical Review Board in Umeå (Dnr 2013/409-31).

### 2.2. Registry data on diagnoses and medication

We linked data from different registries with the unique personal identification number assigned to each resident in Sweden. We retrieved inpatient and outpatient data from the National Patient Registry to identify individuals that were diagnosed during a 20-year time-frame (1993–2013) with CVD (ICD-9: 410-414, 428, 430-436, 444; ICD-10: I20-I25, I50, I60, I61-I64, I67.9, I11.0, I70.2, I73.1, I73.9, I79.2, G45) or diabetes (ICD-9: 250; ICD-10: E10.5, E11.5, E14.5, E11.5). In addition, we retrieved data from the National Diabetes Registry on individuals with diabetes (ICD-10: E10.5, E11.5, E14.5, E11.5) that were treated exclusively in primary care. We acquired data from the Swedish Prescribed Medicine Registry on patients that had filled prescriptions for statins (ATC-code C10A) or antihypertensive medicines (ATC-codes C02, C03, C07, C08, and C09) between 01 January 2013 and 31 December 2013.

### 2.3. Defining the need for primary prevention of cardiovascular disease

To determine the need for medication, we used the myocardial infarction (MI) prevalence as a proxy for CVD risk for physicians and non-physicians. The rationale behind this is that MI prevalence reflects the distribution of CVD risk factors in a given population. On a population level, the use of CVD-preventive medication should be proportional to the distribution of CVD risk factors. [21] We calculated the prevalence of MI (ICD-9 codes 410-414 and ICD-10 codes I20-I25), in one-year intervals, for different sex- and age groups.

### 2.4. Excluded individuals

**Figure 1.**
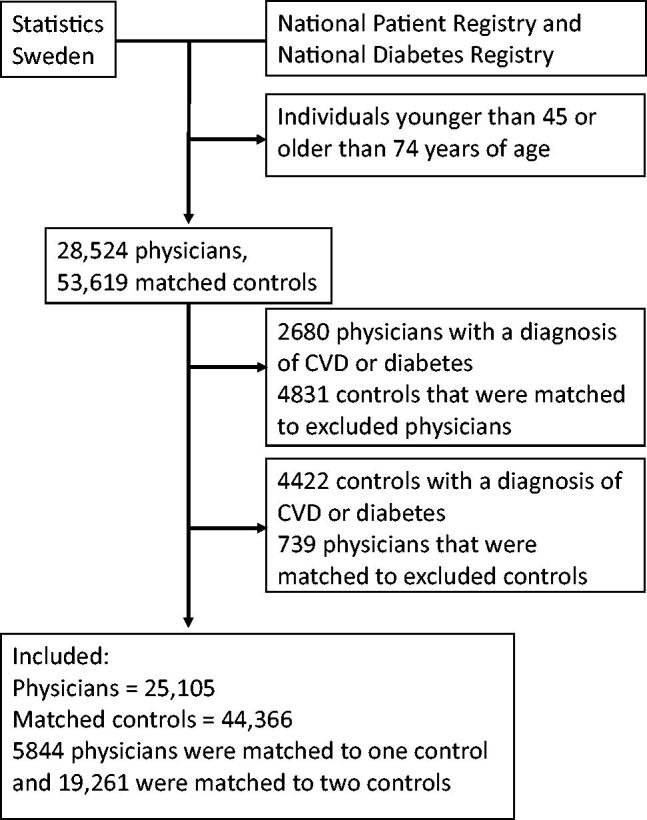
Flowchart of the population selected for the calculation of the main outcome.

After defining the need for cardiovascular preventative measures, we excluded all individuals with existing CVD or diabetes from further analysis. Then, we excluded all physicians that lacked a reference individual (Figure 1).

### 2.5. Covariates on disposable income

Statistics Sweden provided data on the mean annual disposable income for each individual and their household, between the years 2005 and 2013.

### 2.6. Outcomes

The outcome was defined as filling a prescription for preventive medication at least once in 2013. We chose one instance because we did not aim to measure adherence to medication. Treatments were considered equitable between groups when medication for each group was proportional to the estimated group need.

### 2.7. Statistics

Age and mean annual disposable incomes are presented as the means and standard deviations (SD), and proportions are presented as percentages (%). We performed the independent-sample *t*-tests to evaluate between-group differences in the means, and we performed Chi^2^ tests to evaluate between-group differences in proportions. We performed conditional logistic regression to estimate the odds ratio (OR), which indicated the likelihood that a physician would receive medication, compared to a non-physician. After a univariate assessment, we performed a multiple variable regression with a model that included continuous measures of the mean disposable income for individuals in 2005–2013 and the mean disposable incomes of other family members in 2005–2013. To estimate adjusted ORs, the model included sex- and age-stratified MI prevalences as potential confounders. All statistical analyses were performed with the statistical software R.

## 3. Results

### 3.1. Prevalences of MI and diabetes

In the analyses of all physicians and non-physicians, before excluding those with existing disease, physicians had a higher prevalence of MI compared to non-physicians (Table 1). The difference in MI prevalence, which served as a proxy for the need for preventative CVD treatment, was only significant between women physicians and non-physicians. However, the diabetes prevalence was lower among physicians than among non-physicians (Table 1). The diabetes prevalence was more than twice as high among men than among women, but it was similar between the physicians and non-physicians of both sexes.

**Table 1.** Prevalences of excluded disease diagnoses among physicians and non-physicians.

Excluded disease	All		Women		Men	
Myocardial infarction	% (*n*)	*p*-value	% (*n*)	*p*-value	% (*n)*	*p*-value
Physicians	4.3 (27,286)	<0.001	2.3 (11,397)	0.001	5.7 (15,889)	0.083
Non-physicians	3.8 (51,574)		1.8 (22,891)		5.4 (28,683)	
Diabetes						
Physicians	3.7 (27,469)	<0.001	2.2 (11,407)	0.006	5.4 (28,683)	<0.001
Non-physicians	4.6 (51,136)		2.7 (22,676)		6.1 (28,460)	

### 3.2. Characteristics of physicians and non-physicians included in the outcome analysis

After excluding physicians and reference individuals with diagnosed diseases, the main analysis included 69,471 individuals, including 25,105 physicians and 44,366 non-physicians (Figure 1). Of the included physicians, 5844 were matched with one reference individual and 19,261 were matched with two reference individuals. Physicians had higher mean disposable incomes compared to non-physicians (Table 2). The mean age among physicians was 58 years, and 44% were women. The mean yearly income of physicians was 160 thousand SEK higher than that of non-physicians, other household incomes were also higher for physicians but to a lesser degree.

**Table 2.** Characteristics of physicians and matched controls.

Characteristic	All			Women			Men		
	Physicians	Non-physicians	*p*-value	Physicians	Non-physicians	*p*-value	Physicians	Non-physicians	*p*-value
Number of individuals	25,105	44,366		10,953	20,767		14,152	23,599	
Age, years; mean (SD)	58 (8.0)	57.4 (8.0)	<0.001	57.3 (7.9)	57.1 (7.8)	0.005	58.5 (8.1)	57.7 (8.1)	<0.001
Income, 100 kSEK; mean (SD)	5.2 (5.9)	3.6 (3.6)	<0.001	4.7 (8.0)	3.2 (3.0)	<0.001	5.5 (3.7)	4.0 (4.0)	<0.001
Median (Q1-Q3)	4.8 (4.0-5.8)	3.3 (2.6-4.2)		4.4 (3.7-5.2)	3.0 (2.4-3.7)		5.1 (4.3-6.1)	3.6 (2.8-4.6)	
Other household income, 100 kSEK; mean (SD)	2.9 (10.1)	2.6 (5.5)	<0.001	3.4 (15.1)	2.9 (7.2)	0.006	2.5 (2.1)	2.2 (3.2)	<0.001
Median (Q1-Q3)	2.5 (0.6-4.1)	2.3 (0.5-3.6)		2.7 (0.3-4.7)	2.6 (0.3-4.1)		2.4 (1.0-3.6)	2.2 (0.6-3.2)	
Dispensed medication									
Any medicine (%)	30.3	20.4		27.7	18.8		32.3	21.7	
Statins (%)	8.9	5.8		6.5	4.7		10.8	6.7	
ACE-inhibitors + AT II-blockers (%)	15.6	11.5		12.6	9.2		17.8	13.4	
Calcium antagonists (%)	5.9	5.5		4.1	4.2		7.3	6.6	
Diuretics (%)	5.1	3.5		6.1	3.9		4.3	3.1	
Beta-blockers (%)	14.9	7.3		13.8	7.3		15.7	7.4	
Other antihypertensives (%)	0.5	0.2		0.2	0.1		0.7	0.3	
Any antihypertensive (%)	27.4	18.3		25.3	17.0		29.0	19.5	

Among physicians, 30.3% received any treatment, compared to 20.4% of non-physicians, and this difference was similar for women and men (Table 2). Compared to non-physicians, a higher proportion of physicians received treatment with all the medications, except calcium antagonists. The proportions of physicians and non-physicians that received calcium antagonists were similar among women (4.1 vs. 4.2%), and although different among men (7.3 vs. 6.6), the proportional difference was smaller than the proportional differences observed for other medications among men (Table 2).

### 3.3. Odds of physicians receiving treatment compared to matched non-physicians

**Figure 2.**
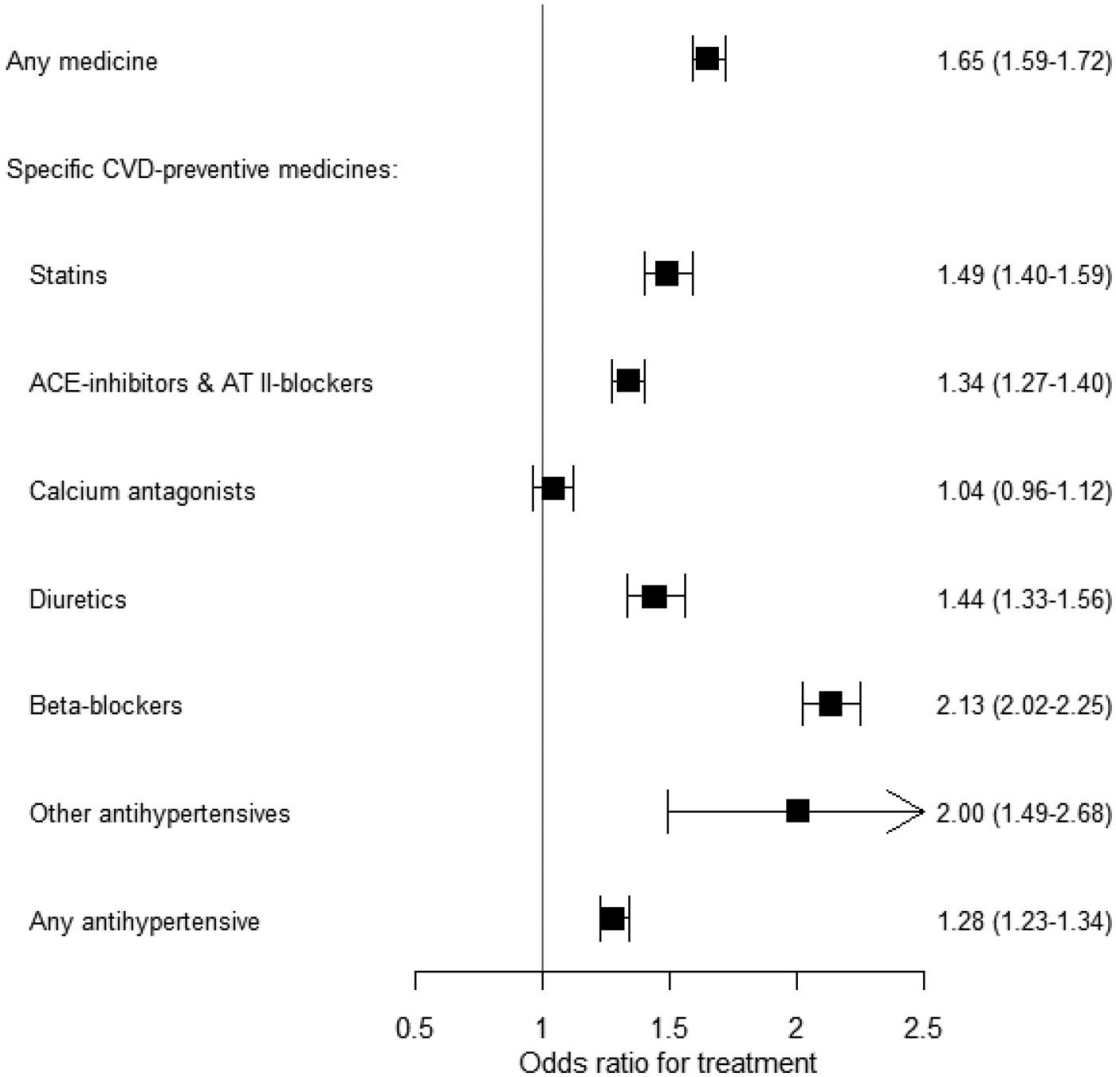
The likelihoods that physicians will receive treatment, compared to non-physicians.^1^ Values are odds ratios and (95% confidence intervals).

The overall OR of receiving any medication was 1.65 (95% CI: 1.59–1.72) for physicians compared to non-physicians (Figure 2). The odds of physicians receiving specific CVD preventative medication compared to non-physicians were similar for any hypertensive medicines (OR: 1.28) (Figure 2). The unadjusted ORs did not differ significantly from the adjusted ORs; for example, for any medication, the unadjusted OR was 1.68 (95% CI: 1.61–1.74) (Supplementary material figure S1).

**Figure 3.**
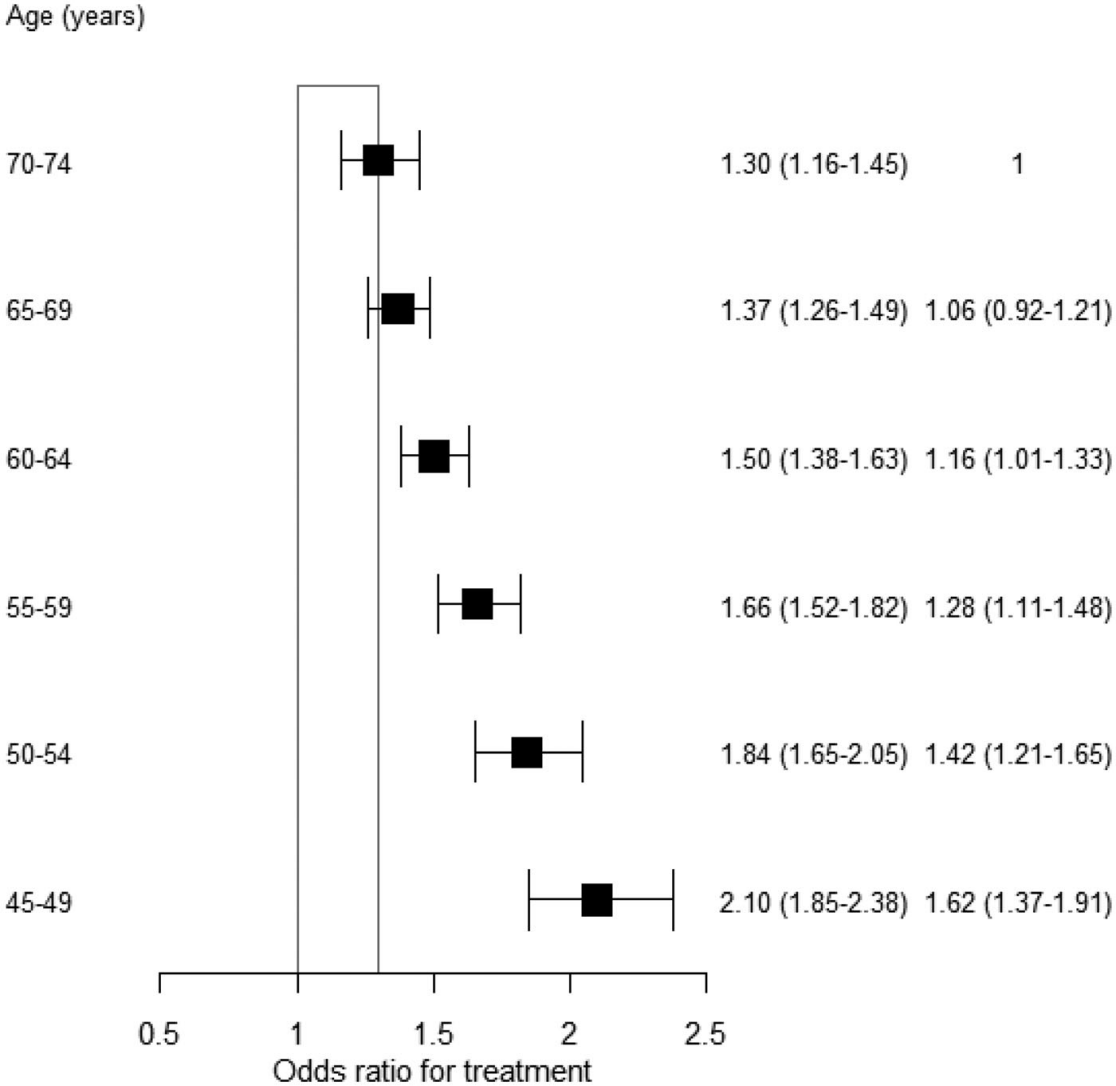
The likelihood that physicians will receive treatment, compared to non-physicians, is stratified by 5-year age groups.^2^ Values are the odds ratios (95% confidence intervals) and the interaction odds ratios (95% confidence intervals).

The OR for physicians receiving any medication compared to non-physicians decreased with age. This OR was 2.1 (95% CI: 1.85–2.38) for ages 45–49 years, and it decreased to 1.3 (95% CI: 1.16–1.45) for ages 70–74 years (Figure 3). This interaction with age was not significantly different between women and men (data not shown). Similarly, the OR decreased with increasing MI prevalence. It decreased from 2.16 in the first quartile to 1.45 in the fourth quartile of MI prevalence (Supplementary material figure S2). In sub-analyses, the ORs for male and female physicians receiving any medication compared to non-physicians were similar (Supplementary material figures S3-S6). However, the OR of physicians receiving diuretics compared to non-physicians was lower among men than among women.

## 4. Discussion

### 4.1. Summary

To our knowledge, this study was the first to highlight data on horizontal inequity between physicians and non-physicians in the use of statins and antihypertensives for primary CVD prevention. One interpretation of the results might be that physicians evaluated risks for themselves differently than they evaluated risks for non-physicians. Another interpretation could be that a greater proportion of physicians had their elevated CVD risk diagnosed, compared to non-physicians. Physicians work within the health care services and this could plausibly lead to higher utilization of health-care. Generally, the data on physicians' health behaviour is of poor quality, for instance, retrospective self-reporting and lack of data among non-responders [22].

That physicians have a tendency to self-treat has been replicated across different cultures [13, 23,24]. This raises the question of whether physicians' proximity to healthcare services translates to elevated use of those services compared to non-physicians. Also, due to physicians having higher trust in medical treatment [25] and medical guidelines [26] than non-physicians, it is likely that a higher share of physicians also accepts medical treatment for themselves when indicated. Because physicians in Sweden can prescribe medicines to themselves the threshold for treatment with medicines may also be lower compared to the more normal situation when one must first consult a physician before a prescription can be obtained.

### 4.2. Strengths and limitations

Compared to other studies on medication use among physicians that were based on self-report questionnaires, we were able to use register data on dispensed medications and thus reduced a potential source of bias. A strength of our study was the use of MI prevalence as a proxy for medication need. This proxy assignment rested on the assumption that the need for preventative medication should be based on total CVD risk, rather than on isolated measures of cholesterol or blood pressure. This assumption was consistent with current guidelines for treatment [11]. The existing risk-scoring charts tend to overestimate CVD risk in high-SEP groups and underestimate CVD risk in low-SEP groups [27,28]. Thus, registry data on actual morbidities are likely to provide a more accurate estimation of group-level risk than a pool of individual risk estimations. Moreover, Swedish registry-based information on MI and stroke is considered valid in-hospital information [29]. Another strength was that we matched highly educated individual controls to physicians, which contributed to a reduction in residual confounding.

The main study limitation was that we did not have access to individual data on blood pressure, cholesterol levels, smoking status, or other traditional CVD risk factors. However, due to the use of MI as a proxy for treatment needs, this limitation would likely not have significantly impacted our results.

Another potential limitation of our study is whether our data from 2013 is generalizable today. Paradigms in preventive medication might have changed since 2013, however, on a national level in Sweden there has not been a paradigm shift in the last decade in how the healthcare system identifies and medicates individuals with elevated CVD risk. Furthermore, there has not been a shift in public discourse regarding CVD prevention.

Clinical guidelines are updated continuously and have an effect on what treatments physicians recommend and when. We however consider it unlikely that they would have a clear impact on non-physician patients’ propensity to seek medical advice regarding their individual CVD risk or on non-physician patients' interest in accepting recommended treatment.

A focus on family physicians would have reflected the prevalence of CVD-preventive medication among the physicians mainly responsible for prescribing these medicines. It is possible that family physicians have a greater interest in CVD prevention compared to the general community of physicians. However, restricting our scope to in such a way would have been a limitation because a physician's speciality does not likely affect their likelihood of being in need of CVD prevention.

### 4.3. Comparison with existing literature

A study from Denmark calculated that 15% of the general population ages 40–75 would be eligible for medication with statins according to the guidelines from the European Society of Cardiology [30]. In our study 8.9% of physicians and 5.8% of non-physicians used statins. Considering that our study population was on the high end of the socioeconomic distribution we would expect that a lower proportion than 15% would be eligible for statin medication.

Our results were consistent with those from a previous study on hormone replacement therapy where physicians were found to have a higher rate of medication compared to non-physicians [14]. However, instead of using the general population as a reference, we selected a reference group with education levels similar to the level required for physicians. Thus, our groups were expected to have similar CVD-risk factor profiles and healthcare behaviours, and thus, potential residual confounders were mitigated. Previous studies that investigated self-prescribed medication among physicians were based on self-reports, and they lacked control groups [13,16,18]. A Norwegian study reported that 10% of physicians aged 50–69 years used antihypertensives [13]. In our study, 27.4% of physicians aged 45–74 years used antihypertensives. This discrepancy might be explained by the possibility that physicians may have underreported their use of antihypertensives in self-report studies. In questionnaire studies, participation biases might also explain the differences in findings. Two other studies found that 1–3% of physicians in their early careers used statins or antihypertensives [16,18]. However, most doctors in their early careers are at an age where antihypertensive and statin treatments are rare. Therefore, we might expect a lower rate of antihypertensive use compared to the physicians in our study. Another study conducted in the US found that, among individuals in the general population aged 40 years and older, 11% had used statins in the last 30 days [31]. In contrast, we found that 8.9% of physicians had used statins. This discrepancy might be explained by the fact that we only included physicians that used statins for primary prevention, and the US study included individuals that used statins for both primary and secondary prevention. Data are scarce on the CVD-risk factor distribution among physicians. However, one study found lower rates of smoking and overweight status among medical students, compared to the general population [32].

This study was not designed to explain the reasons underlying the observed differences in the rates of dispensed medication. Potential explanations could be that physicians might deviate from clinical guidelines or that a greater proportion of physicians are diagnosed to have elevated CVD risk, compared to non-physicians, or a combination of the two. However, we lack scientific evidence on the access physicians have to health care, and the existing studies have presented poor-quality data [22]. Future studies might interview physicians that have prescribed primary preventive medication to themselves to provide a deeper understanding of their decisions. It has been shown that the decision to measure a patient’s blood pressure and cholesterol, for instance, is affected by whether that patient’s physician has undergone such measurements [33].

## 5. Implications for research and/or practice

We found that physicians were more likely than non-physicians to use antihypertensive or cholesterol-lowering medication for primary CVD prevention. This study does not allow for conclusions about the reason why these differences exist. In the case that our findings represent a difference in the discovery of elevated CVD risk then implementing a population-based screening programme for CVD risk is likely to reduce the differences found in this study [34]. In the case that our findings represent a form of deprivation among non-physicians then an intervention to improve adherence to treatment guidelines could be helpful.

## Supplementary Material

Supplemental Material

## Footnotes

1 Adjusted for personal income, family income and the age- and sex-stratified MI-prevalence

2 Adjusted for personal income, family income and the age- and sex-stratified MI-prevalence
