# Supplementary material for: Are doctors using more preventive medication for cardiovascular disease? A Swedish cross-sectional study
Source: Scand J Prim Health Care. 2023 Jul 19;41(3):297–305. doi: 10.1080/02813432.2023.2234439 (PMC10478618; doi:10.1080/02813432.2023.2234439)
Supplement: Supplemental Material [file IPRI_A_2234439_SM6149.docx]

Supplementary material


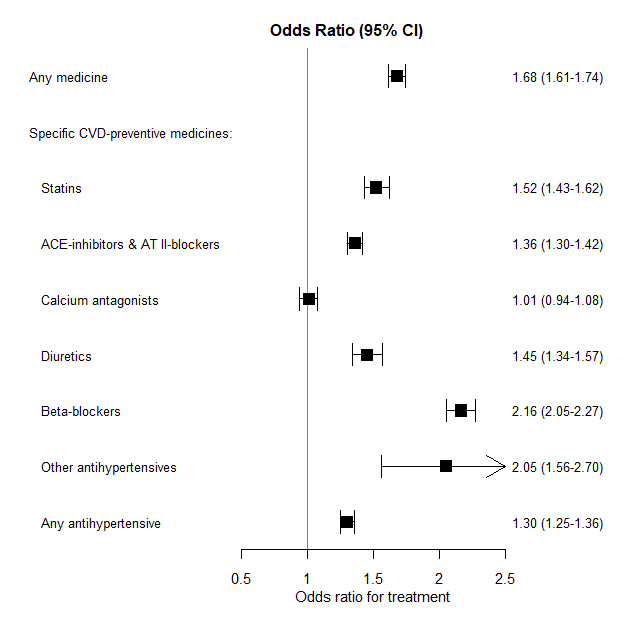


Figure S1. Unadjusted ORs for treatment.


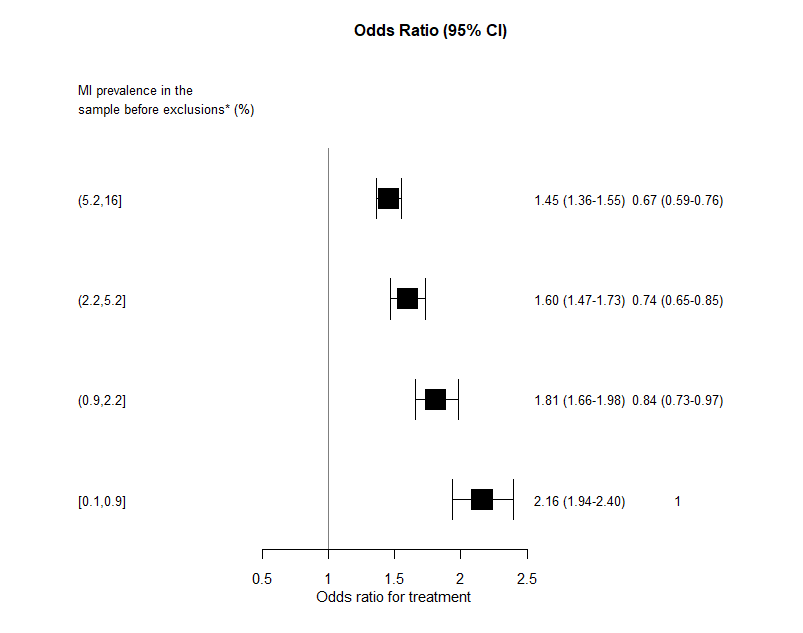


Figure S2. Adjusted ORs for treatment in quartiles of MI prevalence. The right column reports interaction ORs.


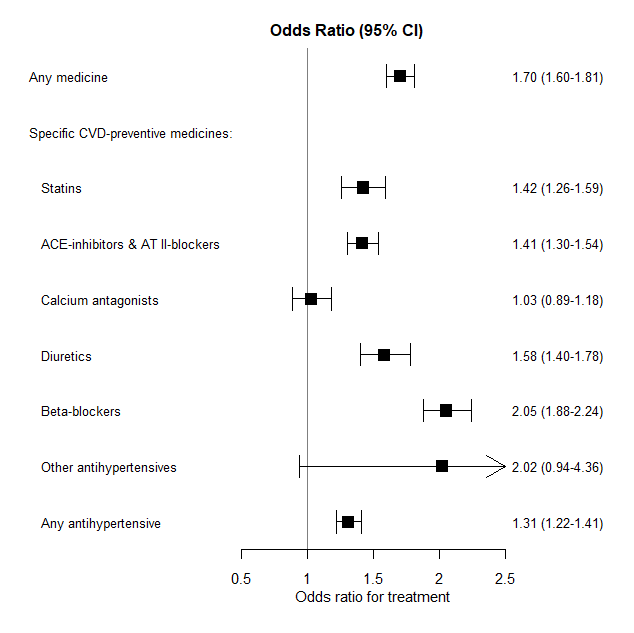


Figure S3. Adjusted ORs for treatment among women.


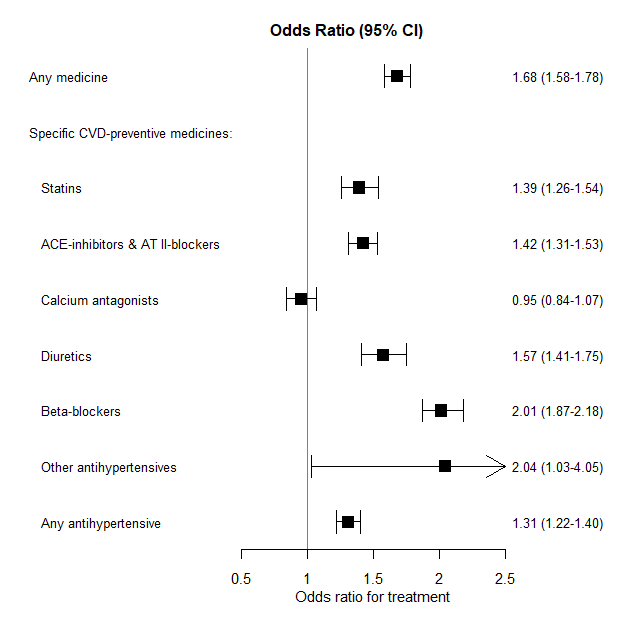


Figure S4. Unadjusted ORs for treatment among women.


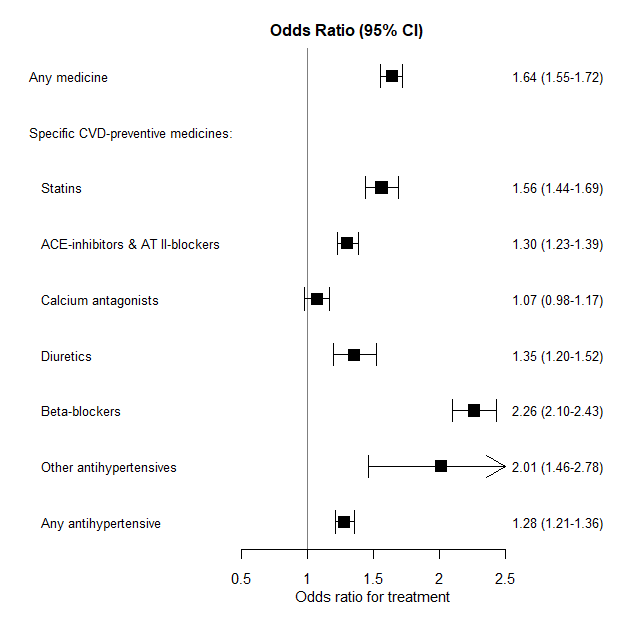


Figure S5. Adjusted ORs for treatment among men.


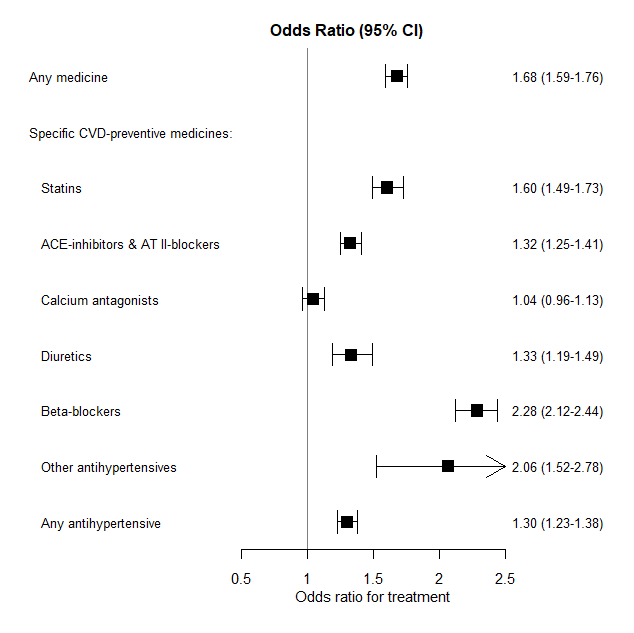


Figure S6. Unadjusted ORs for treatment among men.
